# Socioecologies in shaping migrants and refugee youths’ sexual and reproductive health and rights: a participatory action research study

**DOI:** 10.1186/s12978-024-01879-x

**Published:** 2024-09-18

**Authors:** Michaels Aibangbee, Sowbhagya Micheal, Pranee Liamputtong, Rashmi Pithavadian, Syeda Zakia Hossain, Elias Mpofu, Tinashe Dune

**Affiliations:** 1 School of Health Sciences, Western Sydney University, Penrith, NSW Australia; 2 School of Medicine, Western Sydney University, Campbelltown, NSW Australia; 3 College of Health Sciences, VinUniversity, Hanoi, Vietnam; 4 School of Health Sciences, Faculty of Medicine and Health, University of Sydney, Sydney, NSW Australia; 5 University of North Texas, Denton, USA; 6 University of Sydney, Sydney, NSW Australia; 7 University of Johannesburg, Johannesburg, South Africa; 8 Western Sydney University, Sydney, Australia; 9 Translational Health Research Institute & Diabetes Obesity and Metabolism Translational Research Unit, Western Sydney University, Penrith, NSW Australia

**Keywords:** Migrant and refugee youth, Sexual and reproductive health and rights, Socioecological framework, Multicultural health policy, Digital health, Peer support networks, Migrant health empowerment

## Abstract

**Objective:**

This study explores socioecological factors facilitating the sexual and reproductive health and rights (SRHR) experiences of migrant and refugee youth (MRY) in Greater Western Sydney, Australia. MRY may be at higher risk for poorer SRH outcomes due to cultural, linguistic, and systemic barriers.

**Methods:**

Using participatory action research, 17 focus groups were conducted with 87 MRY aged 15–29 from diverse cultural backgrounds. Data were analysed thematically, using socioecological framework.

**Results:**

Key facilitators of MRY's SRHR were identified at the microsystem and exosystem levels, including (1) Peer dynamics and support, with friends serving as trusted confidants and sources of advice; (2) Safety and contraceptive choices, highlighting the importance of access to contraception and STI prevention; and (3) Digital platforms for SRHR information access, with online resources filling knowledge gaps.

**Conclusion:**

Findings suggest the need for SRHR interventions to leverage peer support networks, expand access to contraceptive options, and develop culturally appropriate digital resources for MRY. Further research is needed to identify and enhance facilitators across all socioecological levels to comprehensively support MRY's SRHR needs.

## Plain language summary

Migrants and refugee youth often struggle to access sexual and reproductive health information and services in their new countries. This study is an attempt to understand what helps young migrants and refugee maintain their sexual and reproductive health and rights in Greater Western Sydney, Australia. Our aim was to identify the positive factors in their environment that make it easier for them to access and use sexual health resources. We talked to 87 migrants and refugee youth aged 15–29 from various cultural backgrounds, conducting 17 group discussions about their experiences with sexual health. Our main results show three important factors that help these young people. The results were, that (1) Many young people trust their friends for advice and information about sexual health, (2) Having choices about contraception and ways to prevent sexually transmitted infections was important, and (3) The internet, especially social media and search engines, is a major source of sexual health information for young people. Understanding these helpful factors can guide better support for young migrants and refugees. It shows sexual health programs need to use peer support in sexual health programs, make sure young people can easily access contraception and protection and create trustworthy online resources about sexual health that are culturally appropriate. Our findings show more research is needed to find other ways to support young migrants and refugees with their sexual and reproductive health. This will help create better health services and education programs for these young people.

## Background

In the evolving field of sexual and reproductive health and rights (SRHR), the specific experiences and needs of migrant and refugee youth (MRY) have become increasingly. In this study, migrants are defined as individuals who have moved to Australia from another country for economic, educational, or humanitarian reasons. MRY refers to young people aged 16–26 who were either born overseas or have at least one parent born overseas. This diverse group encompasses various cultural backgrounds, migration experiences, and levels of acculturation. Similar studies have included first and second-generation migrants, refugees, and international students [1, 2]. MRY face unique challenges in navigating SRHR due to multiple intersecting factors. These include migration status, length of time in the host country, language proficiency, acculturation level, socioeconomic status, and cultural beliefs about sexuality and health [1, 3]. These factors significantly impact MRY's understanding, approach, and utilization of SRHR services in their new environment.

While barriers to SRHR for MRY have been well-documented, including lack of awareness and access to services, cultural and language barriers, and conflicting values between home and host cultures, there remains a notable gap in understanding the positive influences or 'facilitators' that assist MRY in maintaining and protecting their SRHR. [1, 2, 4]. For example, newly arrived refugees may face language barriers and unfamiliarity with the healthcare system, while second-generation migrants might experience cultural conflicts. Additionally, young women from certain backgrounds may encounter barriers due to gender norms. These barriers often intersect with the facilitators explored in this study. Qualitative studies on youth’s SRH, such as Tirado, Chu [5] and Chattu and Yaya [6], have begun to shed light on these barriers. The barriers are compounded by service providers' often inadequate understanding of MRY’s specific needs, leading to insufficient support [1, 2, 4, 7, 8]. Cultural stigmas and misconceptions and lack of formal SRHR education further complicate the landscape, as MRY may rely on informal networks for SRH information, highlighting a crucial gap in formal support systems [4, 9].

In Australia, the homogeneity of SRH education across different regions adds to these complexities. In Greater Western Sydney, where this study is focused, MRY constitute a significant proportion of the population [10]. Studies highlight the inconsistencies in educational content and social pressures and their impact in discouraging MRY from seeking essential SRH information and support [8, 11]. Despite these obstacles, MRYs often exhibit adaptability and resourcefulness, turning to the Internet and media to fulfil their SRH needs [12, 13]. This adaptability demonstrates their resilience and emphasises the need to understand the facilitators in their ecosystems that enhance their SRHR agency and decision-making. However, there is a lack of clear research on how MRY in Western Sydney identify and operationalise these facilitators or fully comprehend and utilise the existing assets in their environments to enhance their perception and agency and empower their SRHR.

This study addressed these gaps by identifying and focusing on the facilitators of SRHR for MRY, particularly exploring how various factors within their socioecological systems contribute to their SRHR knowledge and practices.

## Socioecologies and MRY’s SRHR: theoretical lens

Socioecologies refer to interconnected layers of influence—individual, microsystem, mesosystem, exosystem, and macrosystem — that shape MRY’s experiences and choices [14]. These constitute the key facilitators or barriers at the different levels, contributing to the resilience and adaptability of MRY in their SRHR journey.

At the individual level, factors such as resilience, adaptability, and resourcefulness are posited as potential enablers for MRY to navigate complex SRHR circumstances despite facing barriers like language difficulties, cultural dissonance, and limited knowledge of SRHR services [1, 15–17]. Although highlighted separately in different studies, the interplay of age, gender, and acculturation strategy in the new country with these personal attributes is also considered, influencing their capacity to access and utilise SRHR resources [5, 15, 18]. The microsystem and mesosystem involve MRY's immediate social networks, including family, friends, and school communities. This study will investigate how these interpersonal relationships potentially serve as sources of information and support in the context of SRHR. The dynamics within and between these systems are likely to have significant implications for MRY's ability to manage their SRHR needs [19–21]. At the exosystem and macrosystem levels, the research will consider how broader societal and institutional factors, such as community organisations, healthcare services, and cultural norms, might act as facilitators to accessing SRHR services. This paper explored the role of these broader systems in MRY’s ability to navigate SRHR challenges [5, 22].

**Fig. 1:**
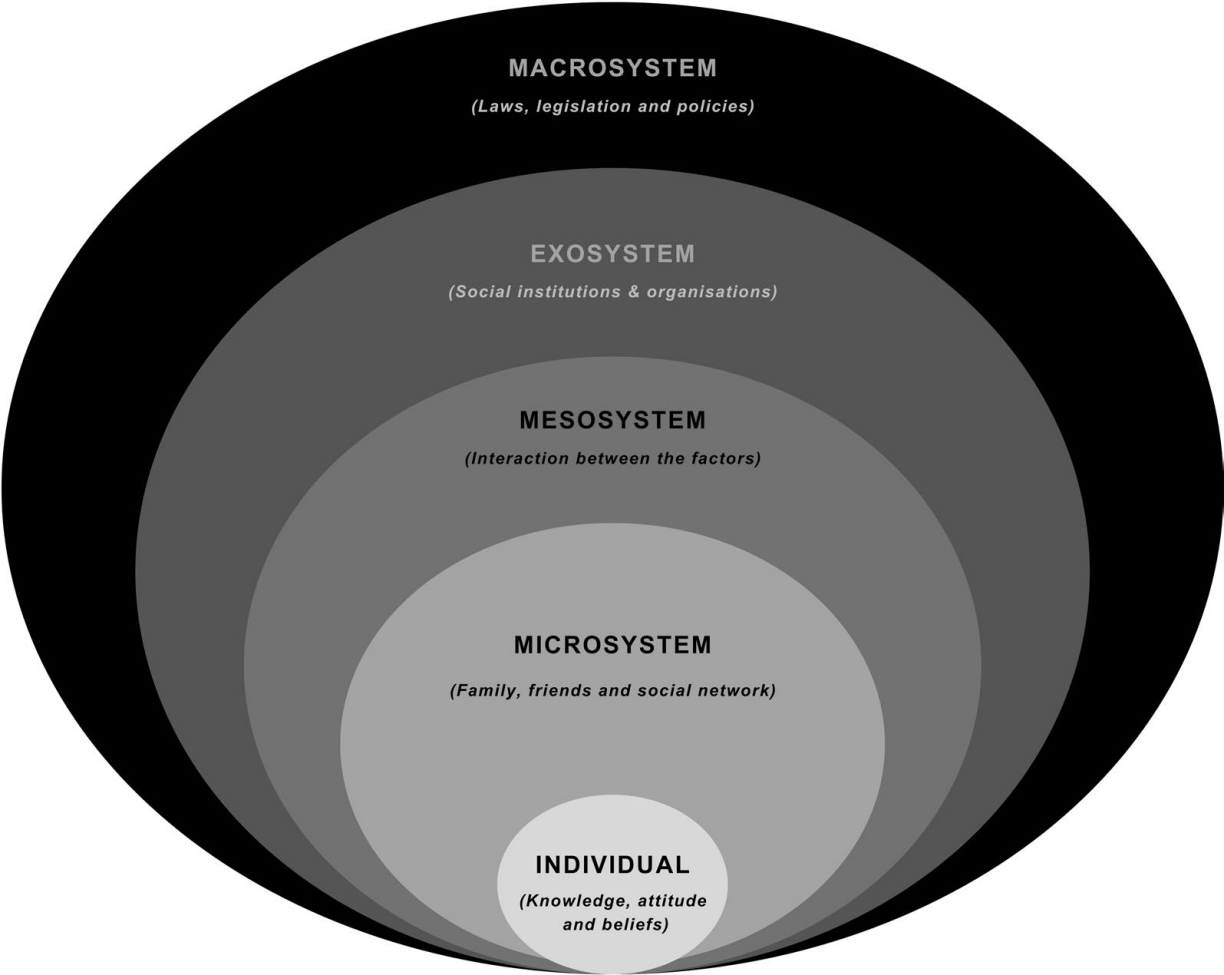
Bronfenbrenner’s socioecological framework

This paper highlighted the nuanced and multifaceted facilitators within MRY's ecosystem using the socioecological framework (Fig. 1). It sought to understand how MRYs, despite the numerous barriers in accessing SRHR support and their decision-making, might employ strategies and leverage available resources to manage their SRHR. This exploration is vital for identifying ways to strengthen these enablers and enhance the resilience of MRY in navigating their SRHR, contributing to effective support strategies and interventions.

## Background of the current study

This study was conducted in Greater Western Sydney (GWS), a region in Australia notable for its cultural diversity and significant migrant population, which comprises 50% of its residents [23, 24]. The choice of Western Sydney is strategic due to its status as a microcosm of global migration trends, characterised by a mix of various ethnic and cultural backgrounds and a dynamic socioeconomic landscape. In 2016, New South Wales had the largest overseas-born population in Australia, with 2,805,971 people (34.8% of the overseas-born population). Sydney, in particular, is a major hub for migrants, with 63.3% of the overseas-born population residing in a capital city, compared to 36.7% of people born in Australia [25]. According to the 2021 Australian Census, approximately 60% of Sydney's overseas-born population lives in GWS, the setting for this study. GWS is renowned for its cultural diversity, hosting over 170 different migrant communities and accounting for about 30% of Australia's total overseas-born population, highlighting its significance as a centre for migrants in Australia [25]. GWS' rapid growth and urbanisation present unique public health challenges, particularly in SRHR, reflecting the complexities of healthcare access and cultural sensitivity in service provision. Studying SRHR in this context offers insights into how cultural norms, health behaviours, and socioeconomic factors intersect to influence the health outcomes of migrant populations. This setting provides a rich context for understanding MRY socioecological factors for SRHR and can inform targeted interventions and policies that are relevant to this region and applicable to other diverse and evolving urban areas worldwide [19].

This paper is based on the second part of a bifocal study primarily focusing on socioecological factors affecting MRY’s SRHR. The first part focused on identifying barriers to MRY's access, decision-making, and utilisation of SRH services [8], while this study examines the facilitators and positive influences on their SRHR journey. Understanding these barriers is crucial for contextualising the facilitators identified in the current study. The barriers and facilitators are often interconnected, and this relationship is further highlighted in Table 3 (see Findings section below) which provides a comprehensive comparison of barriers and facilitators across different levels of the socioecological framework. The qualitative nature of this study is pivotal for its capacity to delve into the depth, nuances, and complexities of MRY's SRHR experiences, capturing aspects that are often overlooked or unattainable in quantitative research. Through this exploration, the research endeavours to offer actionable strategies, informed by a deep understanding of MRY perspectives, to improve their access to SRH services and foster a supportive environment tailored to their needs and circumstances. This research is guided by the following questions:

1. What socioecological factors act as facilitators of SRHR for MRY?
2. How do the identified socioecological facilitators enhance the agency, decision-making, and wellbeing of MRY with regard to their SRHR?

## Significance of the current study

This research is of considerable importance in the field of SRHR, particularly for MRY in Australia, as it identifies and understands the facilitators influencing their SRHR. By addressing a critical gap in existing research, the study highlights the positive factors and resources MRY utilise to navigate their SRHR. Using a socioecological framework and participatory action research (PAR), the study examines how elements within MRY's social and environmental contexts enable their SRHR decision-making and service access.

The significance lies in its focus on facilitators, an underexplored area in SRHR research for MRY, offering fresh insights and a nuanced understanding of how MRY interact with SRHR resources and support systems. This research both contributes academically and has practical implications for policy and service delivery, guiding the development of culturally safe, context-specific SRH services for MRY. Additionally, the findings have the potential to improve community health in Greater Western Sydney and offer global relevance for enhancing SRHR among MRY populations worldwide.

## Method

### Research design

This study utilised a socioecological framework and a participatory action research (PAR) methodology, focusing on the active engagement and empowerment of migrant and refugee youth (MRY) in the research process. PAR is known for its emphasis on the active participation and collaboration of communities in research, particularly effective in health research for addressing complex social issues [26, 27]. In SRHR research, employing PAR necessitates creating a space where research participants, researchers, and project advisory committee members collaborate as equal partners. In this way, they all contribute to defining key issues, co-creating solutions, and implementing changes while ensuring confidentiality and respecting the diverse backgrounds of participants. This methodology allowed research participants to participate actively in shaping research questions, data collection, and analysis, providing essential insights for the study. Bronfenbrenner's socioecological framework was used to analyse and interpret the data, categorising SRHR facilitators for MRY across different levels of influence. This approach helped in systematically examining how factors at each socioecological level interact to shape MRY's SRHR experiences.

### Participants and procedure

The recruitment for this study was conducted in stages commencing 1 June 2020 to 12 June 2021, with the aim of including MRY participants in the research development (Table 1). The study engaged 87 MRY with varying migration backgrounds, and from diverse racial, ethnic, religious, socioeconomic, educational, sexual, and geographical backgrounds, comprising youth project liaisons (YPL, *n* = 8) and MRY (*n* = 79). Demographic data was collected from 75 participants, with a majority identifying as female (*n* = 56, 65.12%) and a minority as male (*n* = 19, 22.09%), aged between 15 and 29 years. Participants were eligible if they were between 16 and 26 years old, as per the ethics approval (Table 1). However, a 15-year-old nearing their 16th birthday, with parental consent and informed assent, and a 29-year-old who met all other criteria were also included. These deviations were considered to enhance the study’s comprehensiveness while maintaining ethical integrity. Appendix A shows that 50.67% of participants were born in Australia, while the remainder were born in countries such as Nigeria, Fiji, New Zealand, Thailand, and Iraq. This sample included both first-generation migrants (overseas-born) and second-generation migrants (Australia-born with at least one migrant parent), capturing a range of experiences with different levels of acculturation and connection to their migrant heritage. The sample represented children of economic migrants, refugees, and potentially expat families, though specific visa categories were not collected.

**Table 1:** YPL-Led SRHR focus group overview and participant criteria

Aspect	Details
Focus group description	Each session, lasting about 60 min, aimed to explore the participants' understanding and experiences related to SRHR, focusing on identifying both barriers and facilitators
Inclusion criteria	Eligible participants were: • Aged 16 to 26 years • Self-identified as migrants or refugees, (with a least one parent born overseas) • Residents of Greater Western Sydney for at least 12 months

Advisory Committee Members (ACM), selected from community leaders, health professionals, and workers in community-managed organisations, contributed to implementing the PAR framework and co-facilitated the recruitment of YPL and MRY in collaboration with the research team. Central to the study, MRY provided qualitative data on their SRHR experiences based on the focus group probe questions (Appendix B). MRY were recruited through various channels, including ACM and YPL referrals, social media, community organisation newsletters, and postings in public spaces across Western Sydney University campuses and Western Sydney. This approach ensured the inclusion of a diverse range of youth in the study.

### Data collection

We conducted seventeen focus groups (*M* = 5.12 participants per session, with YPLs functioning as co-researchers and contributing as participants), predominantly via Zoom® to facilitate participation during the COVID-19 pandemic from 11 November 2020 to 12 June 2021. Using focus groups was important for vulnerable young people as it allowed the participants to interact with each other in a safe environment. It has been used extensively in research involving vulnerable people [26, 28]. In this study, "vulnerable young people" refers to MRY and their potential vulnerabilities regarding SRHR. These vulnerabilities stem from cultural differences, language barriers, unfamiliarity with the healthcare system, and conflicts between home and host culture values. Unlike other young people, MRY face added complexities in accessing SRHR information and services due to their backgrounds. The focus group sessions, primarily held in participants' preferred spaces, ranged from 60 to 90 min, with an average duration of 60 min. The first author co-facilitated 15 out of the 17 focus groups and the fourth author co-facilitated the remaining two sessions. These focus group discussions aimed to explore MRY's understanding and experiences regarding SRH, their rights, challenges, facilitators, and the solutions to address SRHR gaps in Western Sydney. The probe questions (Appendix B) were methodically crafted to explore the depth and breadth of MRY's experiences and understanding. To ensure the diversity of experiences and accessibility, the sessions were held in various formats. While focus groups were not specifically organised by demographic characteristics due to recruitment challenges with MRY populations, natural clustering occurred. This resulted from purposive sampling, where participants often brought in friends or acquaintances through word of mouth or community organisations. Three focus groups were conducted face-to-face to complement the online discussions. Two of these sessions occurred simultaneously in different areas of a local community-managed organisation in Greater Western Sydney, providing diverse and accessible settings for participant engagement.

### Ethical consideration

The study received approval (H13798) from the Western Sydney University Human Research and Ethics Committee (HREC), ensuring ensuring adherence to ethical standards. Participants provided written informed consent and verbal consent at the start of each data collection session, with voluntary participation and the option to withdraw at any time. Confidentiality and anonymity were prioritised, with personal information securely stored on Cloudstor and anonymised. A debriefing process offered support for any distress during focus groups. The principal investigator maintained ongoing ethical oversight, promptly addressing concerns. YPLs, peers within the same age group as the MRY, facilitated the focus groups, reducing power differentials and enhancing the study's participatory nature, trustworthiness, and credibility.

### Data analysis

The thematic analysis of the focus group data was conducted by the first, second, and last authors, following Braun and Clarke’s guidelines [29]. This analysis involved sifting through participants' narratives to identify recurring topics and substantial categories that align with the research objectives. To ensure confidentiality, pseudonyms replaced actual participant names. The software Quirkos® was employed to facilitate the identification of topical responses and the emergence of significant categories. Quirkos® is a user-friendly interface that simplifies the process of coding and analysing qualitative data [30]. It aided in coding various aspects of the data, such as word repetition, direct and emotive statements, as well as discourse markers such as connectives, evaluative clauses and intensifiers, following methodologies by Braun and Clarke and Liamputtong [29, 30]. Additionally, YPLs participated in a workshop to learn the basics of qualitative analysis, and two of them collaboratively analysed two of the 17 focus group transcripts in pairs. The codes they generated were integrated into the broader thematic analysis, enriching the study's qualitative data interpretation.

### Findings

**Fig. 2:**
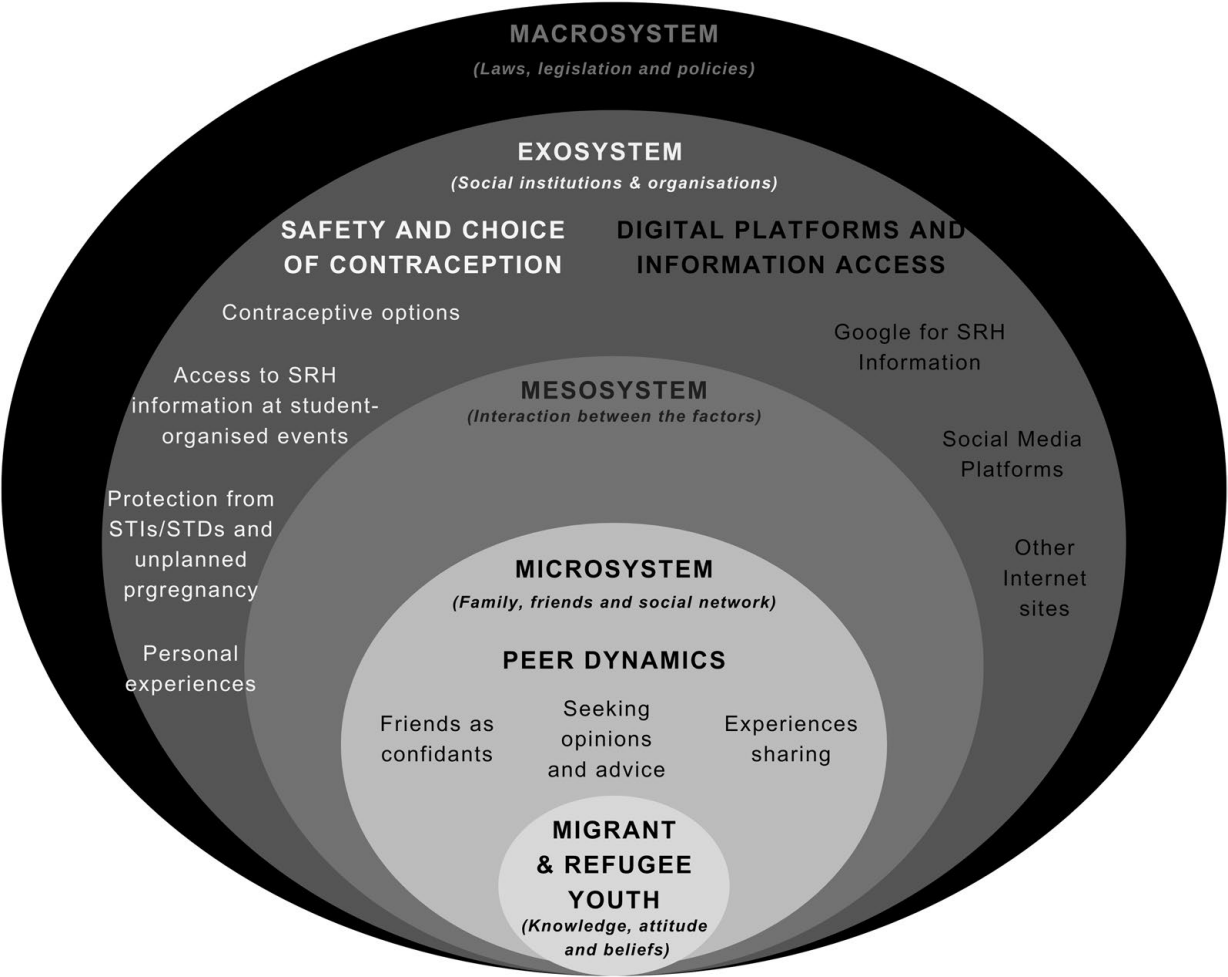
Bronfenbrenner’s socioecological framework: facilitators of sexual and reproductive health and rights in migrant and refugee youth (Sydney, July 2023)

The findings detailed below provide insights into the experiences and perceptions of MRY participants regarding the factors that facilitate the maintenance and protection of their SRHR. Through the thematic analysis of the focus group data, the analysis revealed three key socioecological facilitators of SRHR for MRY that were most relevant to the socioecological model's microsystem and exosystems (Fig. 2). These themes are (1) Peer Dynamics and Support, (2) Safety and Contraceptive Choices, and (3) Digital Information Access, as outlined in Table 2. These findings have also been shown in Table 3 to highlight the barriers and facilitators in the context of the socioecological framework.

**Table 2:** Themes and sub-themes emerging from data

Themes	Sub-themes
Peer dynamics and support	1. Friends as confidants 2. Seeking opinion and advice 3. Experiences sharing
Safety and contraceptives choices	1. Personal experiences 2. Contraceptive options 3. Access to peer SRH information at students organised events 4. Protection from STIs and unplanned pregnancy
Digital and information access	1. Google for SRH information 2. Social media platforms 3. Other internet sites

**Table 3:** Themes highlighting barriers and facilitators under the socioecological framework

Socioecological framework	Barriers	Facilitators
*Individual*		
Microsystems	1. Lack of awareness and access to services, 2. Lack of SRHR education, 3. Fear of fatal consequences, 4. Blame, Guilt and Shame, 5. Alcohol and Other Drugs,	1. Peer dynamics and support a. Friends as confidants b. Seeking opinions and advice c. Experience sharing 2. Safety and contraceptives choices a. Personal experiences b. Contraceptive options c. Access to Peer SRH information at student-organised events d. Protection from STIs and unplanned pregnancy
Mesosystems	1. Family conflict, 2. Social isolation and stigma 3. Gender knowledge gap 4. Sexual violence 5. Lack of confidentiality and trust 6. Insensitivity	
Exosystem	1. Lack of professional support 2. Language barriers 3. Policy impact 4. Lack of SRHR education in the curriculum 5. Lack of access to services	Digital and information access a. Google for SRH information b. Social media platforms c. Other Internet sites
Macrosystem	1. Cultural and societal norms 2. Religious beliefs 3. Moral boundaries 4. Media and culture	

### Microsystem influences

Representing immediate environments and relationships, the microsystem level is evident in the findings on peer dynamics and contraception choices, which highlight the crucial role of close interpersonal relationships and personal decision-making in shaping MRY's SRHR experiences.

### Peer dynamics and support

The data revealed significant insights into how MRY navigate their SRHR through peer dynamics and choices regarding contraception, representing the microsystem level encompassing direct and immediate environments. It revealed subthemes, which include:

*Friends as confidants:* MRY participants frequently reported the necessity of confiding in close friends when addressing SRH issues. This trust-based communication was pivotal in their understanding and decision-making processes. Amongst peers, the power dynamics at play are generally neutral which sets a comfortable engagement as summed up in the following statements among others,

*“I feel like when you're with your friends, you're in a much more comfortable setting…, or for me anyway, I feel like I'm in a much more comfortable setting,” (Adebola, F, 16, Nigerian, FG 17),*

and

*“maybe like you feel more comfortable with your friends because we're all the same age and we're kind of learning this together.” (Kojo, M, 18, Ghanaian, FG 17).*

Another participant added, *“Just the level of trust I have with my friends is really different to my parents” (Georgina, F, 20, Nigerian, FG 17),* emphasising the power dynamics usually present in the parent–child conversation, where the young person is constantly weighing the potential impact of their inquiry. Another stated,

*“When I'm with my friends, they don't seem to be as judgemental or it's not really a topic that we have to think about” (Jana, F, 18, Iraqi, FG 17).*

It reveals that MRY are foremost keen on their peer relationships to explore their SRH concerns before considering other options.

*Seeking opinions and advice:* Advice and opinions from friends, especially those with prior experiences in SRH matters, were crucial in shaping participants' attitudes and approaches towards their SRHR. MRY do this in their bid to make sense of their SRHR situations. A participant stated,

*“Yeah, we talk to friends, and then friends give their experiences, and then we have* [to] *search our own mediums, just trying to understand it.” (Madi, F, 25, Sierra-Leonian, FG 4).*

While participants alluded that their peers’ opinions may not be accurate, it provided the springboard for further exploration, consulting other information channels available to them. Nonetheless, participants agreed that the opinion or advice provided by friends is more likely to be acted upon, with one participant reflecting,

*“So yeah, going to friends would be, would have been my first point in contact to making informed decisions at that time” (Angeline, F, 23, Indian, FG 4),*

*“Yeah, so, like, we learned about it in school for a little bit. And then if I needed further questioning, I had unanswered questions. I guess I would either ask people I knew, like my friends or like the Internet.” (Zantla, F, 18, Bangladesh, FG 2)*.

Although rare, participants also highlighted that their peer group could extend to family members with whom they share similar interests or trust. Lara (F, 19, Indian, FG 4) explained,

*“I think the only person that I felt comfortable talking about it* [sexual problem] *to was one of my cousins, who was actually back home in India.”*

This shows that MRY’s engagement with peer relationships goes beyond their immediate physical environment, provided a shared interest is embedded in mutual trust.

*Experiences sharing:* Conversations with friends who had personally encountered SRH issues provided practical insights and fostered a sense of shared understanding among the youth. Besides family members, participants expressed the ease in having SRH conversations with peers with similar experiences, highlighting that:

*“Except like people our age, like our friends or our siblings [like], and they (*peers) *have as much experience as we do, like we're on the same level” (Merelita, F, 18, Fijian, FG 7).*

*“For me, it's mostly just friends who've done it, and mostly, I mean, if I get to hear something, which I would feel is like, OK, I need to look this up, it'll be mostly Google or again, porn, and it's mostly friends usually. Yeah, those are the main sources I would get information about sex from.” (Navdeep, F, 19, Indian, FG 4).*

From the above, it shows MRY are more likely to solicit the experiences of peers who have had sexual experiences as a guide to making informed decisions.

### Safety and contraceptives choices

*Personal experiences:* The narratives shared by participants highlight the significant impact of personal encounters with SRH issues on their approaches to SRHR. These personal experiences serve as vital pathways for learning and shaping SRHR decision-making. One participant articulated the value of personal agency and the lessons learned from the outcomes of one's choices, stating,

*“It's important to voice your own opinions and draw and then, as a result, have your choices as your actions. And if your actions aren't the best, then you'll have to face the consequences, which is all about the experience” (Johnny, M, 23, Indian, FG 10).*

This reflection indicates that MRYs perceive their capacity to make and act upon decisions, regardless of the consequences, as a fundamental aspect of their SRH decision-making process. Another participant shared their perspective on making informed choices regarding contraception, reflecting,

*“If I was using a pill, if I was using contraception, and even though that, for me, was… the best decision or the most informed decision I could make, and that was like to protect my sexual and reproductive health” (Nadia, F, 21, Polish, FG 4).*

Nadia's account demonstrates how personal decisions about contraception are seen as critical to safeguarding one's sexual and reproductive health. These statements underscore the importance of experiential learning and personal autonomy in the SRHR journey of migrant and refugee youths.

*Contraceptive options:* The data also highlights the impact of the range of contraceptive choices available in facilitating informed decision-making in SRHR. Participants noted that while initially obtaining information and access to contraceptives posed challenges, the availability of various options significantly bolstered their sense of autonomy once these initial barriers were overcome. One participant highlighted the importance of accessible options by recounting,

*“I've actually referred back a lot of my friends to them (*YB Health) *as well, because they used to say, like, oh, I couldn't get access to contraception.” (Peta, F, 22, Chinese, FG 4).*

This indicates the broader community impact of accessible contraceptive solutions beyond MRY. Another participant highlighted the place of accessible contraceptive provisions, sharing,

*“I guess having access to contraception, for one, because obviously I don't want any sort of direct threats, to put it safe, I guess.” (Daniel, M, 21, Australia, FG 1).*

This highlights the empowering effect of having sustained access to contraception, underlining the importance of such services in supporting individuals’ SRHR needs and rights.

*Access to Peer SRH information at student-organised events:* This sub-theme focuses on how university campus events are vital platforms for enhancing knowledge and practices related to MRY’s SRH. The participants highlighted the significance of these events, where free and open conversations about SRH are encouraged, and resources like free condoms are readily available, fostering a supportive environment for safe sexual practices. One participant reflected on the open and informative nature of these gatherings, stating,

*“And like people could just literally go up and ask questions and [the student organisers] were more than willing to talk to you about anything; …that kind of opened my eyes a bit. (Amina, F, 18, New Zealand-Arabic, FG 12).*

This statement illustrates the importance of having a non-judgmental space where young people feel comfortable seeking and sharing information. Similarly, Peta, a 22-year-old Chinese female participant (FG 4), recounted,

*“So while I was at Uni, like my first year, ages ago, we used to talk about this kind of things, sexual and reproductive health, and one of my friends actually referred me to this place called YB Health”*

This narrative highlights the peer-to-peer learning and referral system that can emerge within university settings. Another participant shared her experience of a campus event focused on safe sex, noting the wide array of resources and discussions available:

*“Like a month or two ago, and that was the first time, [like] there was a whole [like] stall on [like] safe sex on campus. I also read for the first time [like] me and my friends, [like wow, like, wow], like, just like it's just out there and [like] they were …like second years and third years [like] explaining stuff like that. There were [like] pamphlets on consent and [like] it was so [like it was like] really integrated well within [like] the week that we had at Uni. And for [like] many of my friends, it was the first time we ever [like]… I'm just using the word out in the open” (Amina, F, 18, New Zealand-Arabic, FG 12).*

Her enthusiastic recount demonstrates the impact of these events in demystifying and normalising discussions around SRH, making them more accessible and less taboo for students.

*Protection from STIs and unplanned pregnancy:* Participants emphasised the importance of safeguarding themselves against sexually transmitted infections/diseases (STIs) and unplanned pregnancies as a fundamental component of their SRHR. One individual expressed,

*“Using protection is* [not] *just for like really unwanted pregnancies, and you don't want an STI, anything (laughs); that's how I consider it, any form (Melinda, F, 23, Arab-Australian, FG 13).*

This comment reflects a broad understanding of the dual purpose of protection, highlighting its role in preventing pregnancies and guarding against STIs.

The sentiment among participants was that prioritising the use of protection was crucial, irrespective of the type, provided it effectively prevented both unplanned pregnancies and STIs. This perspective underlines a proactive and informed approach to sexual health, acknowledging the dual risks involved in unprotected sex. Another participant further articulated this viewpoint,

*“It's just a smart thing to do. Like, you never know, like there's other forms of contraception, obviously, but I feel like you still want to just make sure, I guess, like something can go wrong all the time and you know, people make mistakes.” (Li, F, 22, Thai, FG 13).*

This statement suggests a pragmatic approach to contraceptive use, recognising its importance in mitigating risks and accounting for human error.

### Exosystem influences

The exosystem level, involving broader social settings that indirectly impact individuals, is reflected in the findings on digital platforms, showing how technology and media environments significantly influence MRY's SRHR information-seeking behaviours.

#### Digital and information access

The significance of digital platforms, especially social media and various online resources, was identified as a crucial exosystem influence on MRY. MRY reflected on their major source of information being internet resources such as health e-magazines, social media groups, and specialised websites to gather information on SRHR, significantly shaping their understanding and decision-making. Participants shared insights suggesting, *“Google search health organisations” (Li, F, 22, Thai, FG 13),* illustrating their reliance on internet searches to find relevant health organisations and information. Further emphasising the role of digital media in filling educational gaps, another participant noted,

*“I had to figure it out, what sex really is, and what consent really is and how to have safe sex via social media instead of education.” (Leila, F, 21, Lebanese, FG 7).*

This comment highlights how MRY are self-educating on critical SRHR topics through online platforms in the absence of formal education. Additionally, the ease of accessing specialised services online was underscored by a participant who shared,

*“Personally, I just went to the sex clinic that I found online” (Shiv, M, 19, Fijian, FG 4).*

This statement reflects the proactive steps taken by MRY to seek out services and information digitally to support their SRHR needs.

*Google for SRH information:* The data revealed evidence that Google serves as a vital tool for many participants seeking SRH information. This preference underscores the significance of readily accessible online resources in bridging SRHR knowledge gaps. Participants expressed a strong reliance on this platform, with one noting, *“OK, so Google is kind of like, saving grace” (Ayelen, F, 17, Nigerian, FG 17).* This sentiment was echoed by another who described Google as a "safe haven" for finding crucial health information:

*“Literally, Google is like your safe haven, you know what to search up or what you need to make sure you're safe and whatever” (Rita, F, 18, Liberian, FG 7).*

The straightforward nature of seeking information was further emphasised with the remark.

*“Go Google it, and you find the information for yourself.” (Nas, M, 21, Indian, FG 7).*

Moreover, the discreet and private aspect of using Google was highlighted as a key advantage, especially when discussing topics that might be difficult or taboo to bring up with parents or peers:

*“That's when people start going on Google. You obviously can't talk to your parents, then with Google, you don't have to talk to anybody, just type until you get answers” (Shaw, M, 22, Thai, FG 7).*

This point underscores the value of Google as an accessible, confidential, and user-friendly resource for MRY to obtain the SRH information they need independently and privately.

*Social media platforms:* The data showed that platforms like TikTok, Twitter, Facebook, and Instagram were commonly utilised by MRY to access SRH information. These platforms offer a wide range of content, including personal anecdotes and media articles, providing users with various perspectives on SRH issues. While participants recognised that these platforms are not always reliable sources of SRHR information due to the potential for misinformation, the influence of social media in this space is undeniable. One participant expressed concern about the reliability of these platforms, stating,

*“I agree, young people do generally reach out to social media platforms, which is not ideal, because, again, there's false information, misleading” (Faith, F, 18, Australian, FG 10)*.

Despite these concerns, the role of social media in disseminating information about physiological functions and other SRH topics was highlighted by another participant, *“I had to learn that (*about SRH) via* social media,”* adding,

*“There is so many things that we didn't know until social media came around and we had to discover on our own (Adebola, F, 16, Nigerian, FG 7).*

This indicates that, for many, social media has been a significant source of discovery and learning in areas where traditional education may be lacking. Another participant echoed the sentiment, acknowledging the extensive influence of social media, particularly among the youth,

“*I completely agree that social media is a huge, like it's huge influence. ...Social media has great potential for learning things and it's kind of more geared towards, especially towards the younger population. (Ron, M, 20, Chinese, FG 12).*

This comment underscores the dual nature of social media as both a vast repository of knowledge and a platform that requires critical evaluation and verification to ensure the accuracy and reliability of the information consumed.

*Other internet sites:* The data also indicate the complex and varied online landscape navigated by participants seeking out a wide array of online sites to gather information related to SRHR, spanning health e-magazines to pornography sites despite credibility and misinformation concerns. One participant remarked,

*“So the first line of defence, I would say, is that they would go online and look at some un-credible sources, sometimes they can do that, but they need to filter out through those sources what is reliable and credible, such as .com.au websites, government websites, and from those they can gain some information about treatment options or, yeah, things like that.” (Aylin, F, 18, Turkish, FG 10).*

Other participants echoed similar sentiments about the educational gap, revealing how individuals often turn to the Internet for information and community experiences they feel are lacking in traditional educational settings. Furthermore, the personal nature of the information sought and shared online was highlighted in the comment, *“A lot of the social media that I research on, is personal experience” (Monique, F, 21, Bosnian, FG 7),* reflecting a trend towards seeking and valuing personal narratives and experiential knowledge in understanding and navigating SRHR.

The study also produced several participant-proffered solutions (Table 4), aligning with the identified themes and sub-themes which will be discussed in a supplementary paper. These solutions offer practical insights into enhancing SRHR education and support for MRY.

**Table 4:** Solutions proffered by MRY participants

Solutions	Details
Intergenerational sexual reproductive health education	1. Workshops and groups 2. Parent-teacher meetings 3. SRHR information sessions within school communities
Strong SRHR support network (cultural, peer, school)	1. Migrants and refugees’ community SRHR Peer liaison groups 2. SRH support workers within the school community 3. SRHR advocates and support teams at community institutions
Knowledge of/and access to SRHR services	1. Information and access to local culturally safe SRHR services 2. Establish an SRH helpline specific to MRY 3. Education and support for existing services on adopting multicultural approach and practices

## Discussion

Situating within the Bronfenbrenner’s socioecological systems framework, this paper explored the socioecological facilitators influencing MRY’s SRHR, and how these factors enhance their agency, decision-making, and wellbeing. Key themes identified include peer dynamics and support, contraceptive options, and digital platforms and information access. The findings predominantly align with the microsystem and the exosystem levels, highlighting the role of immediate relationships and digital platforms in shaping MRY’s SRHR knowledge, attitudes, and behaviours. The interaction between these levels showed digital platforms enhancing peer support. However, the absence of mesosystem and macrosystem facilitators highlights potential intervention areas, such as improving communication between microsystems (mesosystem) and addressing broader cultural and policy factors (macrosystem) to better support MRY's SRHR.

Based on the findings, peer-based communication, rooted in trust, plays a significant role in shaping and guiding MRYs understanding and decision-making [2, 31]. Other studies further support our findings, emphasising youths’ preference for peer-sourced information over formal educational sources, highlighting its approachability and relatability [2, 32]. This preference underscores our findings where MRY rely on their peers for SRHR information, valuing the trust and ease of communication within these relationships. The study also revealed that MRY value peer interactions for their limited social sanctioning and the absence of power imbalances, a contrast to family communications. This aligns with Wight et al.’s observation that peer networks significantly influence health behaviours and attitudes, thereby reinforcing the importance of these dynamics in shaping SRHR understanding and practices [33]. The role of peers extends beyond information sharing to exemplifying positive health behaviours, which can be particularly influential in MRY’s SRHR journey [34]. This peer influence is further shaped by broader socio-cultural factors, such as cultural norms and acculturation experiences, affecting how MRY engage in SRHR discussions. While the findings do not always explicitly mention migration status, the cultural and ethnic factors identified by participants are deeply connected to their experiences as MRY. For example, Lara's (F, 19, Indian) reliance on discussing sexual issues with her cousin in India highlights how MRY often navigate between two cultural worlds—a distinctly migrant experience. Similarly, Ayelen's (F, 17, Nigerian) description of Google as a “saving grace” point to the significance of digital platforms for MRY, who may face language barriers or cultural taboos in accessing traditional SRHR sources. Monique's (F, 21, Bosnian) emphasis on culturally safe contraceptive choices further illustrates how MRY negotiate between different cultural expectations around sexual health. The preference for peer-sourced information over formal education, as expressed by Adebola (F, 16, Nigerian) and Kojo (M, 18, Ghanaian), underscores the need for culturally relevant and linguistically accessible SRHR information for MRY. This nuanced understanding of how cultural and ethnic factors influence SRHR experiences is vital for developing targeted and effective interventions for MRY.

However, the reliance on peers for SRHR information comes with challenges. The risk of misinformation, a key concern in our study, is highlighted in Fantaye et al., Mbarushimana et al. and Mulubwa et al.’s conducted studies showing that while peers are key information sources, they can also propagate inaccurate or misleading content especially those gleaned from the unreliable Internet sources [35–37]. This highlights the need for accurate and culturally safe SRHR education [8, 38]. Providing comprehensive education and enhancing well-informed peer education can help mitigate the risks of misinformation and enhance the reliability of peer-sourced information [39–42]. Sun et al.’s research supports the effectiveness of peer-led interventions in health education [43]. Implementing this strategy could include establishing Peer Liaison Groups (PLG) in various institutions and community settings, such as universities, schools, religious institutions, and health centres. These groups would function as safe spaces or one-stop-shop drop-in centres where MRY can seek SRHR information and support. Strengthening these PLG networks with accurate information and training can strengthen their credibility as sources of SRHR information.

Participants emphasised the importance of access to a diverse range of contraceptive methods, a finding that echoes the sentiments in wider academic literature. The ability to choose from multiple options empowers MRY in their SRHR decision-making highlighting the critical need for accessibility to and comprehensive knowledge of SRHR information [44]. This concept is supported by Handebo's and Rugoho’s studies, which stress the importance of informed choice in contraceptive use and its impact on individual autonomy and health outcomes [45, 46]. Informed choice highlights MRY's ability to decide on the method that suits their individual freedom, psychological health and reproductive rights conditioned on a comprehensive knowledge of contraceptive options including their rights to avoid contraception use [45–49]. A focus on expanding the range of contraceptive options for MRY communities is vital. Practical steps at the community level could include making condoms accessible at no cost in common areas like grocery shops, pharmacies, or discreet counters at newsagents equipped with QR codes linked to comprehensive SRHR information and support. This approach, in line with Auerbach and Smith’s recommendations, should be complemented by providing detailed information to facilitate informed choices [50]. While participants acknowledged the empowerment derived from making personal decisions about contraception, they also recognised that positive outcomes are not always guaranteed, often hindered by a lack of comprehensive SRHR education [8].

**Fig. 3:**
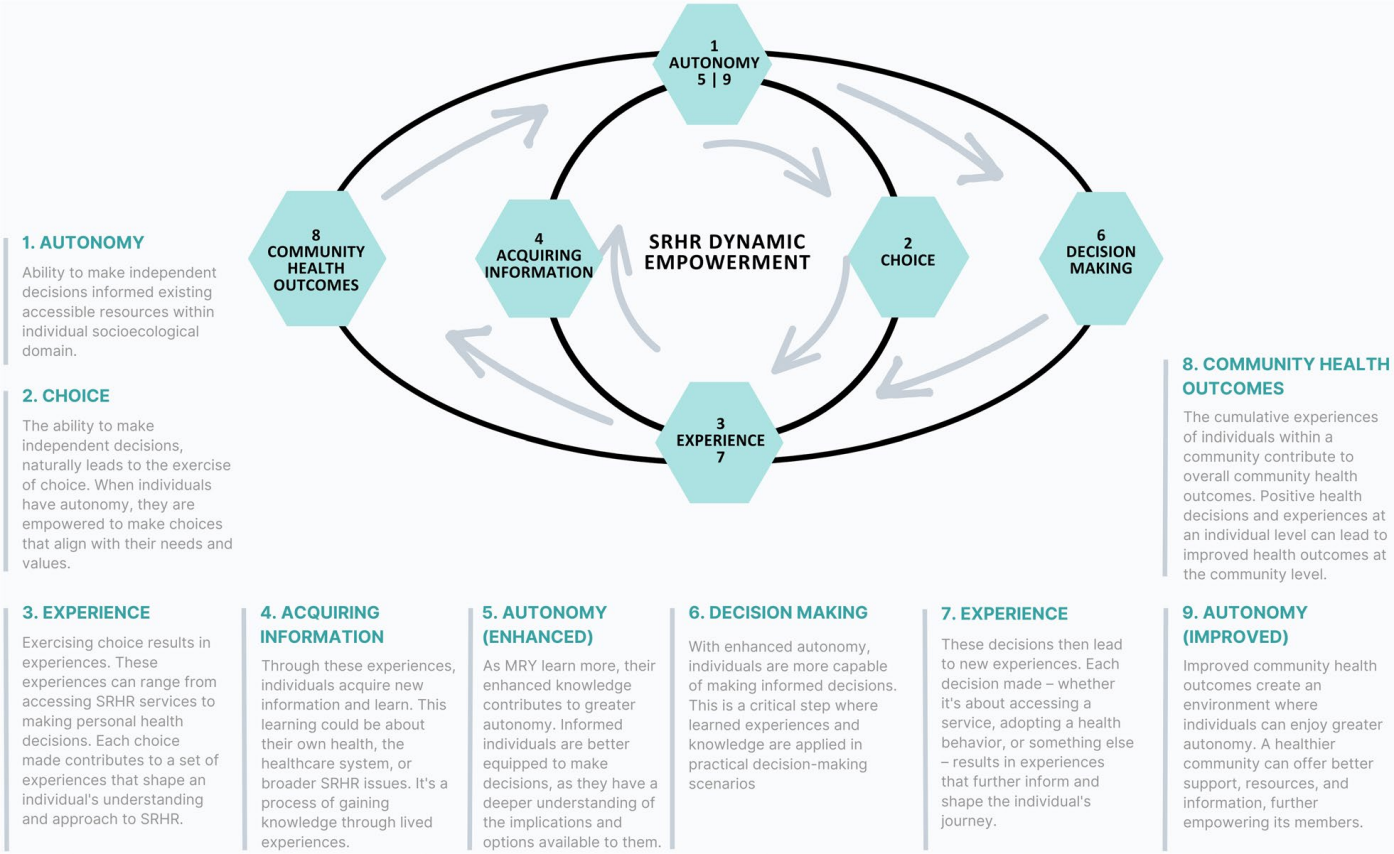
Cyclical dynamics of SRHR empowerment in MRY (Sydney, January 2024)

The study further underscores the fundamental importance of experiential learning and personal autonomy shaping MRY’s SRHR journey, referring to MRY’s agency with their dependence on peer experiences or personal choice around the use of contraception. This finding resonates with the research of Kapitány-Fövény and Kennedy et al., which demonstrate the impact of personal narratives and experiences in health-related decision-making [51, 52]. For instance, MRY’s experiences with accessing and using contraceptives contribute to their understanding and confidence in making future SRHR decisions. This cyclical process, where autonomy enhances experiences, which in turn strengthens further autonomy, highlights the dynamic relationship between personal experiences and autonomous decision-making in SRHR (Fig. 3). This finding aligns with several research works that discussed the positive impact of contraceptive access on individual autonomy and community health outcomes [51–53].

Additionally, the study revealed the value of informal peer-to-peer learning and referral systems, especially in university settings. This pattern of engagement in SRHR information among peers effectively reduces stigma and fosters a supportive community environment, enhancing accessibility to SRHR information and services. Such peer-to-peer interactions are pivotal in promoting a sense of belonging and shared understanding [54]. The study also underscores the importance of student-organized events as platforms for accessible, peer-led SRH information and support [55, 56]. These events serve as vital facilitators in disseminating SRHR information, reflecting the effectiveness of peer-led interventions in addressing the SRHR needs of young populations.

Furthermore, the current study highlights the dual concern of preventing STIs and unplanned pregnancies, a theme that is consistent with broader research in the field. Participants emphasised the critical role of protection in SRHR, reflecting an understanding of the dual purpose of protective measures. This understanding is in line with Træen’s descriptive study in Norway, which reported a significant decline in unplanned pregnancies and STIs among those consistently using protection [57]. Such findings underscore the essential role of effective contraception in safeguarding against both pregnancy and STIs [58]. The study found that participants prioritised protection in their SRHR practices, irrespective of the contraceptive method, as long as it effectively guarded against both unplanned pregnancies and STIs. This proactive and informed approach toward sexual health suggests that access to and utilisation of contraception are key in maintaining SRHR among MRY.

Our findings highlight the crucial role of digital platforms in facilitating SRHR information access for MRY. This aligns with existing research on the increasing use of the internet for health-related information among youth [59, 60]. The preference for digital resources, particularly search engines and social media, reflects a shift in how younger populations, especially MRY, prefer to receive health education [36, 61–64]. These platforms offer diverse perspectives and a broad range of content, facilitating the spread of information and personal narratives that can be instrumental in shaping health behaviours and decisions. Despite concerns about misinformation [65, 66], Burcin et al.’s [63] work suggests that the influence of these platforms in disseminating SRHR information cannot be understated, making them significant facilitators in the SRHR journey of MRY [63].

Moreover, the study stresses how MRY proactively use a variety of online resources, including health e-magazines and other internet sites, to gather SRHR information. This approach aligns with Moss et al. [67] and Nair and Prasanth's [68] findings on the importance of online health resources [67, 68]. The ability of MRY to navigate and filter through these resources for credible information, further emphasises the facilitating role of digital literacy in enhancing access to SRHR information [62, 67, 68]. The pivotal role of digital platforms underscores the need for health educators and policymakers to leverage these platforms effectively. [69]. By acknowledging the growing reliance on digital resources, strategies can be developed to ensure that MRY have access to accurate, reliable, culturally safe, age-appropriate, inclusive and relevant SRHR information. The information should encompass both STI prevention and unplanned pregnancy, emphasising the rights associated with SRH. Leveraging digital platforms such as Instagram and TikTok or other platforms popular among youth can enhance the reach and effectiveness of this education. Improving digital literacy is also essential, considering digital platforms' significant role in SRHR information dissemination. Other studies also support this by highlighting that developing skills to critically evaluate online information is crucial [70, 71]. Additionally, collaborating with digital platform owners and app developers for the dissemination of accurate and engaging SRHR content can maximise reach and impact [71–73].

### Recommendations for theory

Drawing from Bronfenbrenner’s socioecological model, this research project has successfully utilised this lens to investigate the facilitators influencing the autonomy and decision-making in SRHR among MRY [14, 74]. However, to enhance the comprehensiveness of the facilitators of SRHR among MRY, it is essential to adopt a multidimensional theoretical framework that integrates components that specifically address digital literacy, the influence of digital platforms, and the role of peer networks. Incorporating these elements can enrich our understanding of how various environmental systems intersect and contribute to facilitating SRHR knowledge and practices among MRY.

The significant influence of peer dynamics in shaping SRHR information and practices among MRY necessitates the integration of theories centred on peer influence and experiential learning within the socioecological framework. The integration of Bandura's Social Learning Theory [75] (which posits that peer learning occurs through observation, imitation and modelling) and Peer-Led Education models [76] could provide insights into how peer interactions positively impact SRHR decision-making and behaviour among MRY [43, 77]. Additionally, the critical role of digital platforms in disseminating SRHR information suggests the need to incorporate theories related to digital health communication. This inclusion should explore how online environments influence health knowledge and behaviours and the role of digital literacy in health outcomes. The Technology Acceptance Model [78] and the eHealth Literacy Framework [79, 80] could offer valuable perspectives on how MRY engage with digital SRHR resources [79, 81, 82].

Moreover, MRY's diverse cultural backgrounds highlight the importance of integrating cultural and contextual theories. The Cultural Tailoring Framework [83] could be instrumental in enhancing the cultural competence and safety of SRHR interventions, ensuring they are effectively adapted to the unique needs of different MRY communities [84].

### Strengths and limitations

This study, focusing on the SRHR of MRY in Australia’s Greater Western Sydney, offers valuable insights into this demographic's unique experiences. A major strength lies in its diverse participant group, representing various cultural backgrounds. This diversity enriches the study's findings, making them more relevant to a broader audience and enhancing the generalisability of the results. The comprehensive thematic analysis, covering a wide array of themes from peer dynamics to the use of digital platforms, provides a deep understanding of the factors enabling SRHR among MRY. This approach captures the complex interplay of personal experiences, social influences, and information access in shaping SRHR understanding and practices. The study's findings on digital platforms as key sources of SRHR information align with the current information-seeking behaviours of young people, making it highly relevant to modern health education and promotion strategies. This alignment is significant for MRY, who often face challenges accessing traditional SRHR information due to language and cultural barriers. The study's focus on digital platforms reveals how MRY overcome these challenges. Participants used search engines and social media for general SRHR information and to find culturally relevant advice and bridge gaps between home and host cultures. This insight highlights the need for accurate, culturally sensitive online SRHR resources and points to potential avenues for targeted education and outreach programs Additionally, the incorporation of personal and peer narratives lends authenticity to the data, offering a nuanced view of the lived experiences of MRY and the real-life implications of SRHR challenges and strategies.

Despite these strengths, the study has limitations. The reliance on self-reported data introduces the risk of recall bias and social desirability, which may affect the accuracy of participants' responses. The sensitive nature of SRHR topics could lead to underreporting or selective sharing of information, potentially skewing the data. The qualitative nature of this study provides rich, in-depth insights but lacks quantitative data that could offer a broader statistical context to make generalisations. As a part of a larger ARC research grant, a complementary quantitative study is underway, which will address this gap. While the study includes a diverse group of MRY participants, it may not fully capture the experiences of all MRY populations, such as those living with disabilities or belonging to the LGBTQIA community. Cultural, social, and geographical differences, along with individual variations, may limit the applicability of the findings across different MRY contexts.

Overall, this study's use of the socioecological model and the PAR methodology successfully captured the multidimensional SRHR experiences of MRY and provided both strengths and limitations. PAR facilitated a collaborative and empowering process, with MRY participants actively shaping research questions, participating in workshops, facilitating focus groups as YPLs, and contributing to data interpretation, enhancing the cultural relevance and authenticity of the findings. The participatory approach also built trust, leading to richer discussions on sensitive SRHR topics. However, participant involvement varied across research stages, with YPLs less engaged in the final interpretation and write-up due to practical constraints. Despite this, PAR significantly increased the study's trustworthiness by centring MRY voices throughout. Furthermore, future research should aim for a more balanced gender representation and consider the impact of exceptional circumstances, like the COVID-19 pandemic, on study outcomes. Despite its limitations, the study makes a significant contribution to understanding the facilitators of SRHR among MRY, a foundation for further research.

## Conclusion

This study has identified key socioecological facilitators of SRHR for MRY and demonstrated how these factors enhance their agency and decision-making, providing significant insights through Bronfenbrenner’s socioecological model. However, participants mainly identified facilitators at only two levels (microsystem and exosystem). This contrasts with previous research, where barriers were recognised across all system levels. This discrepancy indicates a gap in either the existence or the recognition of facilitators within the other levels of the socioecological system. Our findings prompt important questions about developing and raising awareness of facilitators at all socioecological levels. While barriers to SRHR are acknowledged to span the entire socioecological spectrum, facilitators seem less evident or less recognised by MRY. This gap suggests a need for future research to explore and enhance facilitators within these underrepresented areas. Such research is crucial for achieving a balanced understanding of SRHR for MRY and for developing comprehensive strategies that address both the barriers and facilitators in their SRHR journey.

## Data Availability

The dataset generated and/or analysed during the current study is available under restricted access. The data is located in the Research Data Australia portal at https://doi.org/10.26183/2x5y-v748. For more information or potential collaboration, please contact the corresponding author.

## Appendix A: participant demographics

**Table:**

Variable	n = 75^a^	%
Gender		
Female	56	74.67
Male	19	25.33
Other	0	0.00
Sexual orientation		
Straight	63	84.00
Bisexual	5	6.67
Gay	0	0.00
Pansexual	2	2.67
Asexual	0	0.00
Other or missing^b^	5	6.67
Religion		
No religion	10	13.33
Christian	30	40
Catholic^c^	11	14.67
Buddhist	6	8.00
Greek Orthodox	0	0.00
Islamic	6	8.00
Other or missing^d^	12	16.00
Country of birth		
Australia	38	50.67
Nigeria	7	9.33
Fiji	4	5.33
New Zealand	3	4.00
Thailand	3	4.00
Iraq	3	4.00
Philippines	2	2.67
India	2	2.67
Zimbabwe	2	2.67
Sri Lanka	1	1.33
Italy	1	1.33
Vietnam	1	1.33
Myanmar	1	1.33
Sierra Leone	1	1.33
England	1	1.33
Liberia	1	1.33
Egypt	1	1.33
Bangladesh	1	1.33
Pakistan	1	1.33
Malaysia	1	1.33
	*Mean (Std. dev.)*	*Range*
Age in years	20.02	15–29

^a^At the end of the focus group sessions, participants were asked to complete a Qualtrics survey for additional demographic data collection. Of the 86 migrant and refugee youths participating in the study, 75 completed the survey

^b^Expressions such as 'unsure', 'anything goes', and 'questioning' were reported by participants when describing their sexual orientation. It should also be noted that two participants opted not to share information regarding their sexual orientation

^c^Participants identified with various Christian denominations, including Baptist, Anglican, Pentecostal, Assyrian Orthodox, Coptic Orthodox, and Maronite Catholic

^d^Other religious affiliations reported by participants included Agnostic, Spiritual, and Hindu affiliations. Furthermore, two participants did not report any information regarding religion

## Appendix B: focus group questions

1. What does the term sexual health mean to you?
2. What does the term reproductive health mean to you?
3. What are your human rights in relation to your sexual and reproductive health?
4. What helps you to maintain and protect your sexual reproductive health?
5. What stops you from being able to maintain or protect your sexual reproductive health?
6. What needs to be done differently in Western Sydney to address these SRHR gaps?
